# Breeding strategies for animal resilience to weather variation in meat sheep

**DOI:** 10.1186/s12863-020-00924-5

**Published:** 2020-10-07

**Authors:** Enrique Sánchez-Molano, Vanessa V. Kapsona, Stavroula Oikonomou, Ann McLaren, Nicola Lambe, Joanne Conington, Georgios Banos

**Affiliations:** 1 The Roslin Institute and R(D)SVS, University of Edinburgh, Easter Bush, Edinburgh, EH25 9RG UK; 2 Scotland’s Rural College, The Roslin Institute Building, Easter Bush, Edinburgh, EH25 9RG UK; 3 Department of Genetics, Development and Molecular Biology, Aristotle University of Thessaloniki, University Campus, 54124 Thessaloniki, Greece

**Keywords:** Sheep, Resilience, Climate change, Production, Breeding schemes

## Abstract

**Background:**

The alteration in weather patterns expected due to climate change will affect farm animal performance, probably resulting in lower quantity and quality of available products. A potential mitigation strategy would be to breed selected animals for enhanced resilience to climate change. In this context, resilience would reflect stable animal performance in spite of weather variation. The objectives of this study were to (i) derive and characterise novel animal resilience phenotypes, (ii) investigate their genetic profiles and (iii) assess the impact of integrating them in breeding strategies for genetic improvement in meat sheep.

**Results:**

Random regression models were used to jointly analyse live body weight measured in different time points throughout the growth phases of 4469 Scottish Blackface sheep and weather variables during the same period to derive novel resilience phenotypes. The genetic analysis of these phenotypes revealed significant genetic variance and heritability, and an antagonistic genetic correlation with some animal performance traits. Simulated breeding strategies demonstrated that a relative emphasis of 10% on resilience compared to other traits would enhance performance stability against weather volatility without compromising animal growth.

**Conclusions:**

Novel resilience traits exhibited sufficient genetic variation to be amenable to genetic improvement with selective breeding and are recommended to be included in future breeding goals.

## Background

The effect of human activities on climate change is manifested by increasing average temperatures and seasonal variability [1–5]. Several studies have assessed the impact of these changes on both plant and livestock, predicting a reduction in product quality and quantity as well as an increase in incidence and severity of diseases [6–8].

In livestock, a desirable animal trait would be performance unaffected by climate change. Previous work defined resilience as the ability of farm animals to produce according to capacity regardless of weather variation [9] and reported significant heritability estimates for the new trait. Therefore, selective breeding for enhanced animal resilience may be a potential strategy to mitigate the effect of climate change on livestock production, but resilience phenotypes need to be properly derived based on the particular breeding goal. While the increase of temperatures beyond a certain heat stress threshold will be an important threat in tropical and sub-tropical regions [10, 11], increased weather variation could also pose a strong detrimental effect on animal performance [7, 12] in any climate zone. Therefore, methods to develop resilience-based breeding goals should account for both directionality and variability of climate affecting animal performance.

Random regression models fitting reaction norm functions have been proposed for the study of individual animal performance across a range of weather variables [9, 13]. These functions provide information regarding change in animal performance due to weather change, with a flat reaction norm being indicative of a resilient animal, whose performance will remain unaffected by weather. Descriptors of individual animal reaction norms, such as slopes at particular points of the curve, can then serve as resilience phenotypes. A genetic analysis of these phenotypes would produce estimates of relevant genetic parameters and animal breeding values to be used in selective breeding schemes.

However, before including resilience in future breeding goals, the genetic correlation with other animal traits of interest needs to be examined. Previous studies [9, 14] have shown the existence of an antagonistic genetic correlation between resilience to weather change and production level and, therefore, the simultaneous inclusion of both sets of traits in the breeding goal must be carefully considered.

The objectives of the present study were to (i) derive novel resilience phenotypes associated with meat lamb growth, (ii) investigate the genetic profile of the new traits, and (iii) assess the integration of the new traits in breeding goals and strategies.

## Results

### Novel resilience phenotype description

Descriptive statistics of lamb body weights and weather records are presented in Table 1. Descriptive statistics of ewe traits are given in Table 2.

**Table 1:** Descriptive statistics of lamb body weights during the growth phase and corresponding air temperature

	BW	PreWW	WW	PostWW	FD	MD	CW	T
**Mean**	4	17.2	29.1	40.6	1.5	19.9	18.8	10.26
**Std. dev.**	0.7	3.5	4.6	4.4	0.8	2.4	2.2	4.37
**Minimum**	1.9	6.6	13	25.1	0.1	10.1	10.6	−0.06
**Median**	4	17.2	29.1	40.4	1.4	20.0	18.7	10.52
**Maximum**	6.1	27.1	42.8	60.2	6.2	29.5	26.7	18.99

*BW* birth weight (kg); *PreWW* pre-weaning weight (kg; ca. 50 days old); *WW* weaning weight (kg; ca. 116 days old); *PostWW* post-weaning weight (kg; ca. 237 days old); *FD* fat depth (mm), *MD* muscle depth (mm); *CW* carcass weight (kg); *T* Average air temperature during the 10 days before weighing (°C)

**Table 2:** Descriptive statistics of ewe traits

	AWG	AWW	APostWW	LS	PL
**Mean**	10.19	27.09	40.77	1.31	2.65
**Std. dev.**	0.97	4.12	3.86	0.47	1.17
**Minimum**	6.85	11.80	27.40	1	1
**Median**	10.16	27.28	40.60	1	3
**Maximum**	14.40	42.40	57.60	4	6

*AWG* average individual growth performance of offspring (average gain weight of the offspring in kg); *AWW* average weaning weight of offspring in kg; *APostWW* average post-weaning weight of offspring in kg; *LS* litter size of first lambing of ewe; *PL* productive longevity (number of lambings) of ewe

**Fig. 1:**
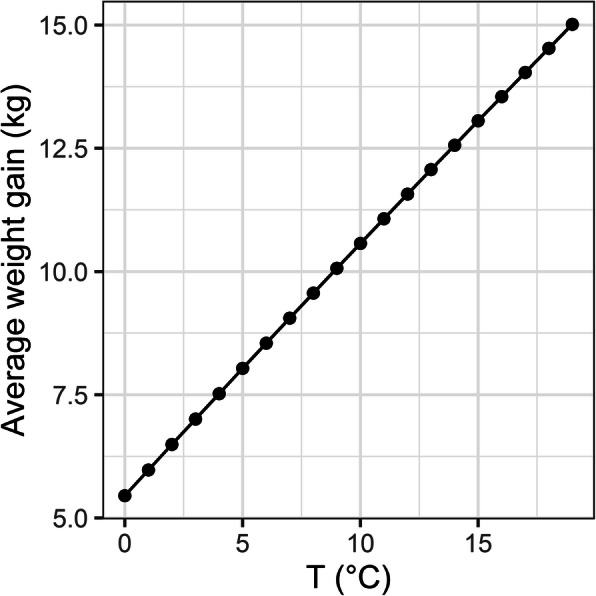
Population reaction norm with non-linear polynomials: Lamb average weight gain change in response to air temperature (T) variability fitting second degree Legendre polynomials

Non-linear random regression models using second degree Legendre polynomials for the derivation of resilience phenotypes were initially explored, revealing a linear trait profile (Fig. 1). Therefore, all subsequent analyses were performed using linear functions with first degree Legendre polynomials.

Descriptive statistics of two resilience phenotypes (growth performance response to weather change and performance stability in changing weather) are shown in Table 3. Individual phenotypic values of response to weather change were both positive indicating an increased weight gain in response to increasing air temperature (and vice versa), and negative indicating a reduced weight gain in response to increasing air temperature.

**Table 3:** Descriptive statistics of two resilience phenotypes

Resilience Phenotype	Mean	SD	Min	Max
**Response**	0.5051	0.1098	−0.0997	0.9211
**Stability**	0.7061	0.0819	0.0309	0.9597

*Response* resilience phenotype reflecting change in average weight gain per unit of air temperature change (°C); *Stability* absolute value of Response (square root transformed); *SD* standard deviation

### Genetic parameters of resilience phenotypes

Variance component and heritability estimates for animal resilience are shown in Table 4. When resilience was considered as a lamb trait, all estimates were significantly greater than zero (*P* < 0.05); however, when resilience was considered as a ewe trait (growth resilience of ewe’s offspring), the stability phenotype analysis did not provide a significant genetic variance estimate.

**Table 4:** Variance component and heritability estimates of two resilience phenotypes

Trait	Resilience Phenotype	V_**P**_	V_**A**_	h^**2**^
**Lamb**	**Response**	9.51E-03 ± 1.85E-04*	1.39E-03 ± 2.65E-04*	0.146 ± 0.027*
	**Stability**	5.27E-03 ± 1.02E-04*	7.29E-04 ± 1.44E-04*	0.138 ± 0.026*
**Ewe**	**Response**	1.21E-02 ± 3.24E-04*	6.86E-04 ± 3.38E-04*	0.057 ± 0.028*
	**Stability**	6.92E-03 ± 1.86E-04*	2.34E-04 ± 1.77E-04	0.034 ± 0.026

*Response* resilience phenotype reflecting change in average weight gain per unit of air temperature change (°C); *Stability* absolute value Response (square root transformed); *V*_*P*_ phenotypic variance; *V*_*A*_ additive variance; *h*^*2*^ heritability; * = significantly different from 0 (*P* < 0.05)

Tables 5 and 6 provide the estimates of genetic and phenotypic correlations amongst the lamb and ewe traits, respectively. Significant antagonistic genetic correlations were detected between weaning weight and both resilience phenotypes (0.711 and 0.671, respectively), implying that genetically heavier animals at that growth phase tend to be more variable in response to air temperature fluctuations. Furthermore, increased response and volatility of animal production to weather change was associated with more muscled and fatter carcasses (Table 5).

**Table 5:** Correlation matrix for lamb traits

	WG	WW	PostWW	FD	MD	CW	Response	Stability
**WG**	0.303*	0.719*	0.981*	0.052	0.092	0.876*	−0.103	− 0.152
**WW**	0.458*	0.276*	0.756*	0.279*	0.328*	0.504*	0.711*	0.671*
**PostWW**	0.988*	0.510*	0.320*	0.074	0.093	0.879*	0.027	−0.016
**FD**	0.139*	0.416*	0.149*	0.143*	0.284*	−0.054	0.443*	0.425*
**MD**	0.164*	0.492*	0.184*	0.362*	0.433*	0.181	0.494*	0.481*
**CW**	0.772*	0.275*	0.785*	0.163*	0.224*	0.192*	−0.122	−0.165
**Response**	−0.254*	0.672*	−0.180*	0.335*	0.398*	−0.155*	0.146*	0.997*
**Stability**	−0.271*	0.646*	−0.200*	0.318*	0.386*	−0.172*	0.991*	0.138*

Heritabilities of lamb traits presented on the diagonal (bold), genetic correlations above the diagonal and phenotypic correlations below the diagonal. Standard errors ranged from 0.0004 to 0.140. *WG* average individual growth performance (average weight gain); *WW* weaning weight; *PostWW* post-weaning weight; *FD* fat depth; *MD* muscle depth; *CW* carcass weight; *Response* resilience phenotype reflecting change in average weight gain per unit of air temperature change (°C); *Stability* absolute value of Response; * = significantly different from 0 (P < 0.05)

**Table 6:** Correlation matrix for ewe traits

	AWG	AWW	APostWW	LS	PL	Response	Stability
**AWG**	0.098*	0.471*	NC	0.558*	0.186	0.037	0.030
**AWW**	0.297*	0.193*	0.471*	−0.384*	−0.067	0.717*	0.755*
**APostWW**	NC	0.297*	0.098*	0.558*	0.115	0.068	0.092
**LS**	−0.027	−0.248*	−0.027	0.098*	0.079	−0.491*	−0.628*
**PL**	0.034	0.072*	0.039*	−0.048*	0.215*	−0.140	−0.130
**Response**	−0.147*	0.430*	−0.168*	−0.113*	0.095*	0.057*	0.999*
**Stability**	−0.147*	0.416*	−0.189*	−0.109*	0.092*	0.989*	**0.037**

Heritabilities of ewe traits presented on the diagonal (bold), genetic correlations above the diagonal and phenotypic correlations below the diagonal. Standard errors ranged from 0.016 to 0.320. *AWG* average individual growth performance of offspring (average gain weight of the offspring); *AWW* average weaning weight of offspring; *APostWW* average post-weaning weight of offspring; *LS* litter size of first lambing of ewe; *PL* productive longevity (number of lambings) of ewe; *Response* resilience phenotype reflecting change in average weight gain of the offspring per unit of air temperature change (°C) Stability = absolute value Response; * = significantly different from 0 (P < 0.05); NC=Non-estimable due to failure to converge

Estimated accuracies of animal Estimated Breeding Values (EBVs) were computed as the square of the average reliability with individual reliability being one minus the ratio of the prediction error variance to the trait variance. Accuracies ranged from 0.43 to 0.55 for lamb traits and from 0.26 to 0.41 for ewe traits.

### Assessment of breeding strategies

**Fig. 2:**
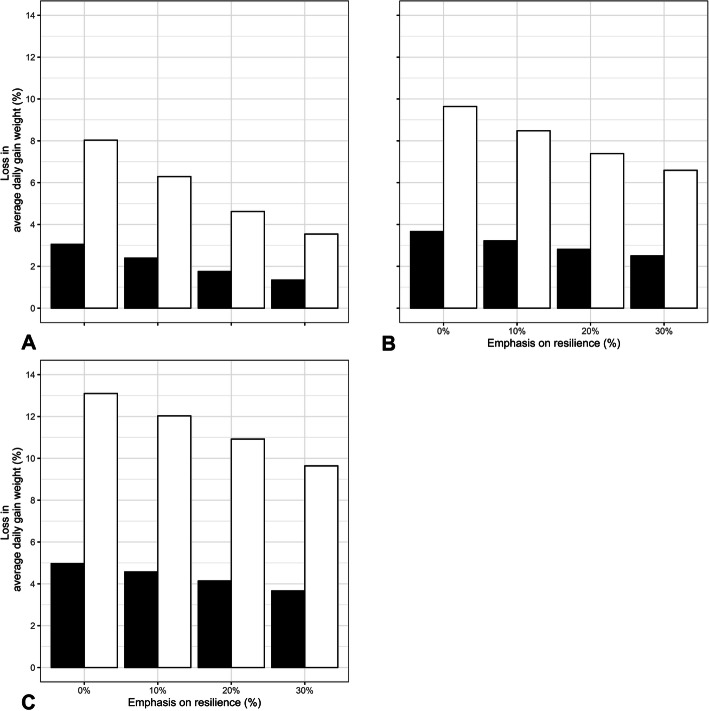
Predicted loss in performance due to temperature: Predicted loss in daily body weight gain after 20 generations of selection by level of emphasis (X axis) placed on resilience defined as lamb growth performance response to weather change trait (**a**) and stability to weather change (**b**) or resilience defined as average ewe offspring growth performance response to weather change (**c**); black and white bars represent temperature changes of 1^o^ and 2.6 °C, respectively

Results of simulated breeding strategies including resilience as lamb traits are presented in Tables 7 and 8. Selection emphasis placed on each trait was chosen to maximise progress within biological boundaries. In these strategies, when resilience was not included in the breeding goal, weather volatility associated with an air temperature change of 1 °C led to an average reduction in growth potential of the lambs by 3–4% (Table 7). Furthermore, the impact of weather volatility associated with a temperature change of 2.6 °C, which according to some predictions is the expected air temperature increase by the end of the century [15], led to increased losses in growth potential up towards 8–10%. When resilience accounted for 30% of the breeding goal, these losses were reduced by more than half (Fig. 2a and b). However, the antagonistic correlation of resilience phenotypes with other traits implied a reduction on the overall animal performance by at least 6% when emphasis on resilience was higher than 20%.

**Table 7:** Breeding for enhanced resilience defined as growth performance response to weather change in lambs

Strategy	Selection Index weights (%)						G	Average per generation								Losses (1 °C)	Losses (2.6 °C)
	WW	PostWW	FD	MD	CW	Res		WW	PostWW	FD	MD	CW	Res	ADG	OPerf		
**Base**	5	5	60	15	15	0	0	28.698	41.460	1.491	19.878	18.844	4.954	0.158	2.393	3.97%	10.46%
							20	38.053	52.286	1.505	24.407	23.258	4.842	0.202	3.358	3.05%	8.03%
**1**	5	5	50	15	15	10	0	28.698	41.460	1.491	19.878	18.844	4.954	0.158	2.393	3.97%	10.46%
							20	36.616	52.601	1.496	25.067	23.590	3.823	0.203	3.424	2.39%	6.29%
**2**	5	5	40	15	15	20	0	28.698	41.460	1.491	19.878	18.844	4.954	0.158	2.393	3.97%	10.46%
							20	34.442	52.022	1.484	25.530	23.543	2.783	0.202	3.433	1.75%	4.62%
**3**	5	5	30	15	15	30	0	28.698	41.4560	1.491	19.878	18.844	4.954	0.158	2.393	3.97%	10.46%
							20	32.212	50.804	1.475	25.439	23.159	2.083	0.197	3.360	1.34%	3.54%

Results are presented for the initial (0) and final (20) generations (G). Simulated traits are: *WW* weaning weight (kg); *PostWW* post-weaning weight (kg); *FD* fat depth (mm), *MD* muscle depth (mm); *CW* carcass weight (kg); *Res* resilience as performance response to weather change ((kg/^o^C)×10); *ADG* average daily weight gain (kg/day); *OPerf* phenotypic index combining all traits but not resilience. Losses in average daily weight gain due to weather changes are presented for 1^o^ and 2.6 °C temperature change. Base strategy does not include resilience, while other strategies have an increasing emphasis on it

**Table 8:** Breeding for enhanced resilience defined as growth performance stability to weather change in lambs

Strategy	Selection Index weights (%)						G	Average per generation								Losses (1 °C)	Losses (2.6 °C)
	WW	PostWW	FD	MD	CW	Res		WW	PostWW	FD	MD	CW	Res	ADG	OPerf		
**Base**	5	5	60	15	15	0	0	28.730	41.466	1.496	19.915	18.823	69.835	0.158	2.383	3.91%	10.30%
							20	37.876	52.002	1.512	24.241	23.163	75.955	0.200	3.303	3.66%	9.64%
**1**	5	5	50	15	15	10	0	28.730	41.466	1.496	19.915	18.823	69.835	0.158	2.383	3.91%	10.30%
							20	36.133	52.954	1.499	23.553	23.570	71.943	0.204	3.297	3.22%	8.48%
**2**	5	5	40	15	15	20	0	28.730	41.466	1.496	19.915	18.823	69.835	0.158	2.383	3.91%	10.30%
							20	33.319	52.820	1.489	22.382	23.559	67.079	0.204	3.157	2.81%	7.39%
**3**	5	5	30	15	15	30	0	28.730	41.466	1.496	19.915	18.823	69.835	0.158	2.383	3.91%	10.30%
							20	30.109	51.614	1.467	21.120	23.099	62.576	0.199	2.999	2.50%	6.59%

Results are presented for the initial (0) and final (20) generations (G). Simulated traits are: *WW* weaning weight (kg); *PostWW* post-weaning weight (kg); *FD* fat depth (mm), *MD* muscle depth (mm); *CW* carcass weight (kg); *Res* resilience as stability to weather change ((kg/^o^C)×100, square root transformed); *ADG* average daily weight gain (kg/day); *OPerf* phenotypic index combining all traits but not resilience. Losses in average daily weight gain due to weather changes are presented for 1^o^ and 2.6 °C temperature change. Base strategy does not include stability, while other strategies have an increasing emphasis on it

Results for breeding strategies where resilience was simulated as a ewe trait (growth resilience of the ewes’ offspring) are presented in Table 9. As before, due to the favourable correlations among some traits, selection emphasis weights were chosen to maximise progress within biological boundaries. When resilience was not included in the breeding goal, losses due to weather change after 20 generations were around 5% of the average daily weight gain of the ewes’ lambs per 1 °C of temperature change and 13% per 2.6 °C change. Adding resilience to the breeding goal with an emphasis of up to a 30% would reduce these losses by 3.5% for an air temperature change of 2.6 °C (Fig. 2c). However, while the overall performance remained unaffected when emphasis on resilience was below 20%, increasing the latter over 20% would lead to a reduction of 2–3% in the overall performance, due to the antagonistic correlation between resilience and weaning live body weight of the offspring in the breeding goal.

**Table 9:** Breeding for enhanced resilience defined as growth performance response to weather change in ewes’ offspring

Strategy	Selection Index weights (%)					G	Averages per generation							Losses (1 ° C)	Losses (2.6 ° C)
	AWW	APostWW	LS	PL	Res		AWW	APostWW	LS	PL	Res	ADG	OPerf		
**Base**	10	10	60	20	0	0	27.085	40.765	1.310	2.666	5.027	0.129	4.327	4.93%	12.99%
						20	28.7370	43.650	1.997	3.793	5.161	0.132	5.560	4.97%	13.10%
**1**	10	10	50	20	10	0	27.085	40.765	1.310	2.666	5.027	0.129	4.327	4.93%	12.99%
						20	27.844	43.648	2.001	3.999	4.719	0.131	5.580	4.57%	12.03%
**2**	10	10	40	20	20	0	27.085	40.765	1.310	2.666	5.027	0.129	4.327	4.93%	12.99%
						20	26.827	43.545	2.004	4.132	4.253	0.130	5.585	4.14%	10.92%
**3**	10	10	30	20	30	0	27.085	40.765	1.310	2.666	5.027	0.129	4.327	4.93%	12.99%
						20	25.557	43.261	2.010	4.117	3.727	0.129	5.524	3.66%	9.64%

Results are presented for the initial (0) and final (20) generations (G). Simulated traits are: *AWW* average weaning weight of offspring (kg); *APostWW* average post-weaning weight of offspring (kg); *LS* litter size of first lambing, *PL* productive longevity (number of lambings); *Res* response to weather changes ((kg/^o^C)×10); *ADG* average daily weight gain of the offspring (kg/day); *OPerf* phenotypic index combining all traits but not resilience. Losses in average daily weight gain due to weather changes are presented for 1^o^ and 2.6 °C temperature change. Base strategy does not include resilience, while other strategies have an increasing emphasis on it

## Discussion

The present study set out to investigate sheep resilience phenotypes reflecting the ability of individual animals to continue growing against weather variability caused by climate change. Reaction norm functions were fitted to random regression models to derive novel resilience phenotypes, whose genetic profile and incorporation into breeding strategies were then examined.

Similarly to a previous study on dairy goats [9], reaction norms for meat sheep were found to be linearly associated with air temperature in the temperate Atlantic conditions prevailing at the venue of the present study. However, reaction norms for other weather variables or geographical and climatic zones might not follow linear patterns and exploration of non-linear functions would then be recommended. Nevertheless, the methodology used in the present study to develop resilience phenotypes based on reaction norm slopes is still valid under non-linear assumptions, where different slopes may be estimated at different points of the curve, thereby providing multiple phenotypes of resilience depending on the value of the weather variable.

Two animal resilience phenotypes were examined here, one representing the directional response of the change in growth performance due to weather volatility and another representing the stability of growth performance in changing weather. While both phenotypes can be used effectively to perform selection towards animal growth unaffected by climate change, the use of the stability phenotype described in this study may prove more intuitive to farmers and the industry. Furthermore, the two resilience phenotypes had a genetic correlation close to unity, indicating that both are largely under the same genetic control mechanism.

Genetic correlations of resilience phenotypes with weaning body weight were antagonistic and relatively high in the present study, while correlations with weight in later stages (post-weaning weight) were not significantly different from zero. This difference may be related to the higher vulnerability of younger animals to weather changes and reflective of the complexity of maturation processes that take place during the growth period. Thus, early life weights may likely be more influenced by weather changes. This is consistent with previous studies indicating that lambs at the weaning stage are particularly susceptible to different stressors that can cause a reduction in performance and welfare [16]. Furthermore, in concordance with previous studies [9], heritability estimates for resilience phenotypes were generally low and in line with estimates derived for fitness related traits [17]. Although relatively low, heritability estimates were significantly greater than zero, implying the presence of substantial genetic variation in the animals’ capacity to cope with weather change. This indicates that resilience may be improved with selective breeding within a properly designed genetic improvement programme.

In order to study the impact of including animal resilience in the breeding goals for meat sheep, different breeding strategies were examined in the present study with simulations considering varying levels of emphasis on resilience compared to other important animal traits. Two separate breeding programmes were considered: a terminal programme and a maternal programme, where resilience was treated as a lamb trait and as a ewe trait, respectively, the latter reflecting growth resilience of the ewe’s offspring. In both cases, the impact of 20 generations of selection on animal performance was assessed.

Failing to include resilience in the breeding goal would result in losses in the growth potential of lambs amounting to 4–5% per 1 °C of air temperature change. Since current variation in daily temperature often pertains to changes by more than 1 °C, cumulative losses due to weather volatility may become quite substantial. In the case of the terminal breeding programme, inclusion of resilience in the breeding goal with a relative emphasis of 10% of the selection index had a very small negative impact on animal performance and led to a substantial reduction of losses in lamb growth potential due to weather variation. Further increasing the emphasis on resilience led to reduced progress in weaning weight and muscle depth, due to the antagonistic correlation of resilience with these traits.

In the maternal breeding programme, similar results were observed. Including resilience with a relatively low emphasis in the selection index led to a reduction of the losses in ewes’ offspring growth due to weather change without strongly affecting other traits. In this case, an expected favourable correlation was observed between ewe longevity and lamb resilience, with lambs from long-living ewes being less susceptible to weather change.

In the present study a constant genetic correlation among traits was assumed across generations of selection. While theoretically genetic correlations may change as a result of new linkage disequilibrium arising due to directional selection, the effect would be more pronounced in single-gene or oligogenic traits. Under an infinitesimal model for polygenic inheritance, an equilibrium state is expected to be reached after a few generations of selection, where the new linkage disequilibrium generated by selection is compensated by recombination [18]. Considering the estimates of the genetic parameters for resilience in the present study, a polygenic trait inheritance pattern can be assumed and relatively minor changes would be expected in the genetic correlations due to selection. Therefore, the assumption of a constant genetic correlation is not expected to change the conclusions of the study. Future functional genomic studies of resilience are needed to investigate the genomic architecture and confirm the polygenic nature of the trait.

The expected strength and direction of climate change in the region of interest must be considered when including resilience traits in breeding schemes. When the resilience phenotype reflects the slope of the reaction norm (directional response), positive slopes will indicate a better performance of the animals as temperature increases and a worse performance as temperatures decrease. Similarly, when resilience is measured as the absolute value of the slope (stability), values closer to 0 will indicate a generally stable performance regardless of the changes in environment. In situations where the directional aspect of climate change (such as the increase on the average yearly temperatures) is more important than the increased weather variation, resilience phenotypes based on actual slopes might prove more useful, allowing to select animals with increased performance in the direction of the expected climate change. However, when increased weather instability becomes more important than the directional change, resilience phenotypes based on absolute values of slopes might be chosen in order to select animals that are more resilient to short- and medium-term environmental fluctuations.

The different weather variables used to derive resilience phenotypes should be also carefully considered when performing this type of analyses. In our study, the 10-day average temperature was generally representative of the corresponding average of the growing season. However, other geographic regions may have more variable temperatures or the impact of climate change in those regions may be more related to weather variation than to average change. In these cases, more appropriate weather variables to test could be those related to dispersion of weather values over a wide time interval.

Furthermore, it is important to consider the possibility of multiple resilience phenotypes to accommodate varying levels of climatic change. Although the traits developed in the present study showed almost linear reaction norms, other traits and breeds might demonstrate non-linear behaviours. In this case, individual reaction norms can provide slopes at different points of the curve, thus allowing the selection of multiple resilience phenotypes at different temperature ranges. These resilience phenotypes could accommodate different breeding goals, such as to provide a stable or increased production in low temperatures and to increase or stabilise performance under heat stress. However, under non-linear reactions norms, it is possible that correlations among EBVs of the resilience trait in different weather ranges vary when slopes for those ranges are changed. Thus, when dealing with non-linear reaction norms, it would be important to carefully consider the future implications of climate change and the desired breeding goals, as well as review genetic correlations among animal traits on a periodic basis.

The present study addressed the direct effects of weather volatility on animal growth. However, climate change and weather instability are expected to also increase the risk of certain diseases such as parasitic infections. Increased disease incidence may further challenge animal growth and other important traits. Follow-up research should focus on the generation of appropriate data and studies to address this indirect impact of climate change on livestock performance.

## Conclusion

The present study has demonstrated the utility of random regression models in developing novel phenotypes for resilience of animal growth performance to air temperature variation in a population of Scottish Blackface meat sheep. Heritable genetic variation was estimated for these phenotypes that can underpin their incorporation in future breeding goals. As current breeding strategies for the genetic improvement of small ruminants do not consider such traits, losses in animal performance can be expected due to climate change. A modest emphasis of 10% on these novel phenotypes in the breeding goal and selection index would contribute to minimising future performance losses associated with climate change without compromising other important animal traits.

## Methods

### Data

Animal performance data from a Scottish Blackface sheep farm located in Scotland were obtained, including 17,876 live body weight records of 4469 animals during their growth phase, spanning the period 1991–2018. All lambs were born on the research hill farm of Scotland’s Rural College and managed under extensive grazing conditions with improved and semi-improved grassland areas for lambing [19]. Three different genetic lines are run within this flock. Ewes are mated in single-sire mating groups from mid-November until early January and, therefore, lamb during April and May. After weaning, all lambs are managed in separate sex groups on the improved and semi-improved grazing until October, when they are selected for breeding, with ewe lambs being moved off farm and males housed for finishing. Live body weight data are recorded up to four times on each animal at the following growth stages: birth weight (BW) recorded on each lamb within 24 h of birth; pre-weaning weight (PreWW) recorded at an average age of 50 days; weaning weight (WW) recorded at an average age of 116 days; and post-weaning weight (PostWW) recorded on average at 237 days of age. In addition, ultrasound muscle (1 longissimus depth record) and fat depth (average of 3 records 1 cm apart laterally) measurements are taken at the third lumbar vertebra on the same day as the weaning weight for each lamb.

Using the four live body weight records described above, three weight gain records per animal between consecutive measurements were calculated and used as multiple growth records to estimate the resilience phenotypes.

Pedigree records were also obtained, consisting of 19,908 animals spanning 22 generations. Animals with phenotypic records were progeny of 281 sires and 2986 ewes.

Weather data including daily air temperatures were obtained from the nearest weather station for the same time period and matched to the above data. To account for possible cumulative effects of temperature, each growth record was matched to the average daily temperature in the 10-day period preceding the recording date.

### Derivation of individual animal resilience phenotypes

A random regression model fitting reaction norm functions on air temperature was used to analyse animal growth records [9] using the BLUPF90 software [20] without fitting pedigree information.

The general random regression model including a reaction norm function is:

1 $$ {y}_{ij}=\mathrm{X}+f\left(\beta, {X}_j\right)+{f}_i\left({a}_{i,}{x}_j\right)+{e}_{ij} $$

where *y*_*ij*_ corresponds to the performance record of animal *i*, at a given environment *j*, *X* corresponds to a set of fixed effects describing all environments, *f*(*β*, *X*_*j*_) corresponds to a function (population reaction norm) describing the relationship between average animal performance and environment *j*, *f*_*i*_(*a*_*i*_, *X*_*j*_) corresponds to a function (individual animal reaction norm) describing the relationship between individual animal *i* and environment *j* (expressed as a deviation from the population reaction norm) and *e*_*ij*_ corresponds to the residual. Previous studies on this breed [21] have shown a negligible impact of maternal behaviour on weight gain of lambs. Therefore, maternal effects were not considered in this model.

In the first instance, second degree Legendre polynomials were fitted to explore the linearity of the population reaction norm. Following model examination, further analyses were conducted with first degree Legendre polynomials. Therefore, the simplified model with first degree Legendre polynomials was:

2 $$ {y}_{ij}=\mathrm{X}+\mu +{\mu}_i+\left(s+{s}_i\right)\ast {\mathrm{X}}_{ij}+{e}_{ij} $$

where *μ* corresponded to the population average intercept, *μ*_*i*_ corresponded to the animal *i* intercept deviated from the population intercept, and *s* and *s*_*i*_ corresponded to the population and individual *i* (as deviation) slopes on the fixed effect (environment); all other terms were as in model (1). The population reaction norm then was *μ* + *sX*_*ij*_ and the individual reaction norm was *μ*_*i*_ + *s*_*i*_*X*_*ij*_, expressed as deviations from the population reaction norm.

Fixed effects included in the model were sex (2 levels), genetic line (3 levels), birth-rearing rank (10 levels; birth rank and rearing ranks combined), age of dam at lamb’s birth (6 levels), grazing code (based on grazing area or heft; 16 levels), length of growth stage (number of days between two consecutive weight recordings; covariate) and date of recording (covariate).

Individual animal reaction norms were computed to reflect the average effect of temperature across all growth stages by adding the population reaction norm to the corresponding individual deviation, and slopes of these functions were estimated using derivatives to represent the change in individual animal growth performance due to air temperature fluctuations. Two phenotypes per lamb were derived from the reaction norms: i) the actual slope representing the individual animal response to weather change, indicating directionality of the change in animal performance (weight gain) per unit of air temperature change and ii) the absolute value of the slope representing stability of growth performance in varying weather conditions.

### Genetic parameters of resilience phenotypes

Variance components of both resilience phenotypes (response and stability) were obtained from statistical analysis with mixed models using pedigree information (relationship matrix A) and the ASReml software [22]. The general mixed model fitted to each phenotype follows:

$$ y= Xb+ Zu+e $$

where ***y*** represents the phenotypic observations for all animals, ***b*** is the vector with fixed effects with design matrix ***X***, ***u*** is the vector with random effects with design matrix ***Z*** and ***e*** is the vector of random errors.

The stability phenotypes (based on the absolute value of individual slopes) were first square-root transformed to approximate a normal distribution. In two separate sets of analyses, resilience was considered as either a trait of the lamb itself or a trait of the lamb’s dam (ewe trait). When resilience was considered as a lamb trait, the model of analysis included the fixed effects of calendar year of birth, birth-rearing rank, sex, genetic line, age of dam at lamb’s birth, recording age, birth weight and grazing code, and the random additive genetic effect of the lamb. When resilience was considered as a ewe trait, the model included the fixed effects of genetic line, birth-rearing rank and grazing code of first offspring, calendar year of first lambing and age at first lambing of the ewe, and the random additive genetic effect of the ewe. Only significant effects (assessed using Wald’s test) where included in the models.

Univariate analyses were conducted separately for each of the four resilience phenotypes (growth performance response and stability as lamb and ewe traits) to estimate the additive genetic variance and heritability of the trait. Multi-variate analyses were then performed to estimate genetic correlations between the resilience phenotypes and other traits of interest, with fixed effects and covariates similar to those tested in the univariate analyses. When resilience was analysed as a lamb trait, correlations were estimated with individual average performance (average individual weight gain between consecutive growth stages), weaning (WW) and post-weaning (PostWW) body weights, carcass weight (CW), and ultrasound fat (FD) and muscle depth (MD). For resilience as a ewe trait, correlations were estimated with the average offspring performance (average body weight gain of offspring between consecutive growth stages), average weaning (AWW) and post-weaning (APostWW) body weights of the offspring, litter size (LS) at first lambing of the ewe and productive longevity (PL; total number of lambings) of the ewe.

### Examination of breeding strategies: simulation model

Variance component and genetic parameter estimates for all traits derived above were considered as inputs to simulation studies to assess the impact of genetic selection for enhanced animal resilience to weather change. In these simulations, resilience phenotypes (derived from slopes of reaction norms and their absolute values) were considered as distinct individual animal traits and simulated together with the other traits of interest mentioned above. In these simulations, genetic and residual correlations among traits were assumed to be constant across all generations.

A genetically heterogeneous population of 1000 individuals (500 males and 500 females) was simulated. Animal traits were assumed to follow normal distributions and simulated using a polygenic model consistent with the infinitesimal theory, thus assuming presence of many loci each with small additive effects on the traits [23]. True breeding values of an individual (TBV) and environmental deviations (ENV) in generation zero (i.e. before genetic selection) were simulated for each trait from multivariate normal distributions TBV ~ (0, **G**_**0**_) and ENV ~ (0, **E**), where **G**_**0**_ and **E** are the genetic and residual variance-covariance matrices for the simulated traits, respectively [24, 25]. All matrices were examined to ensure they were positive-definite.

Twenty (20) non-overlapping generations were simulated by selecting the best males (30%) and females (50%) in each generation and mating them randomly to produce offspring. The TBVs of the offspring were calculated as TBV_offspring_ = 0.5(TBV_sire_ + TBV_dam_) + MS_TBV_, where MS_TBV_ represents the Mendelian sampling terms derived for each trait from a multivariate normal distribution MS_TBV_ ~ (0, 0.5**G**_**0**_(1- $\overline{F}$)), with **G**_**0**_ corresponding to the genetic variance-covariance matrix in generation zero and $\overline{F}$ was the average pedigree inbreeding coefficient of the parents [24, 25]. Environmental deviations for the offspring were computed from the multivariate normal distribution described above.

Selection of parents for the next generation was based on the estimated breeding values (EBV), which were computed for each individual and trait assuming an EBV accuracy (representing the correlation between TBV and EBV) consistent with the analysis of actual data described above. These EBVs were standardised and combined into a breeding goal index comprising multiple traits and different levels of emphasis on each trait (selection index weights in Tables 7, 8 and 9). The resulting index was used to rank the animals and select the best ones as parents for the next generation.

Finally, phenotypic values were derived for each animal and trait by adding the respective TBV to the environmental deviation and the phenotypic mean (mean estimated in the current data described above) in generation zero (before selection).

### Examination of breeding strategies: breeding goals and assessment

Two separate breeding programmes were considered: one aiming to improve traits of the final product, meaning the lambs to be fattened and slaughtered (terminal programme) and another aiming to improve maternal traits of the ewes that produce these lambs (maternal programme).

To facilitate computations, response to weather changes and stability phenotypes were scaled by 10 and 100, respectively, because of their relatively small variances compared to other traits.

In the simulated terminal programme, traits included in the breeding goal and their direction of selection were: weaning and post-weaning weight (increase), carcass weight (increase), muscle depth (increase), fat depth (stabilise and maintain constant), and resilience (selection direction towards zero, signifying no change in performance in response to weather change).

In the simulated maternal programme, only the phenotype reflecting response to weather changes was simulated, because the genetic variance of the stability phenotype was found to be not statistically greater than zero. Traits included in the breeding goal and direction of selection were the average offspring weaning and post-weaning weights (increase), litter size of the ewe at first lambing (increase to 1.8 then stabilise), ewe longevity (increase), and resilience (selected towards zero, signifying no change in performance due to weather changes).

In all cases, a base strategy excluding resilience from the breeding goal index was simulated. Subsequently, additional strategies placed increasing emphasis on resilience compared to the other traits (Tables 7, 8 and 9). All strategies were evaluated according to response to selection of each trait separately and all traits collectively. For the latter, all traits other than resilience were combined into a phenotypic index of overall performance by using the same trait weights as the corresponding base strategy (Tables 7, 8 and 9).

An estimate of the average daily weight gain (average animal performance divided by the average interval between successive weightings) was also simulated in each strategy, representing the growth potential of the individual lamb or ewe’s offspring in the terminal and maternal programmes, respectively. This growth potential was not included in the breeding goal but was used in the assessment of the different breeding strategies. After 20 generations of selection, changes in the expected growth potential due to weather change were calculated and assessed by un-scaling the slopes (expected change per degree of temperature on average growth potential in the entire growth stage) and presenting them as percentages of average daily gain change due to weather (assuming a linear daily growth rate within stage).

## Data Availability

Data might be commercially sensitive. For more information, please contact GB.

## Abbreviations

AWW: Average weaning weight of offspring
APostWW: Average post-weaning weight of offspring
BW: Birth weight
EBV: Estimated Breeding Value
ENV: Environmental deviation
FD: Fat depth
LS: Litter size at first lambing
MD: Muscle depth
PL: Productive longevity; total number of lambings
PostWW: Post-weaning weight
PreWW: Pre-weaning weight
TBV: True Breeding Value
WW: Weaning weight

## References

[1] Agovino M, Casaccia M, Ciommi M, Ferrara M, Marchesano K. Agriculture, climate change and sustainability: the case of EU-28. Ecol Indic. 2019;105:525–43.

[2] Lin BB (2011). Resilience in agriculture through crop diversification: adaptive Management for Environmental Change. BioScience.

[3] Rowell DP (2005). A scenario of European climate change for the late twenty-first century: seasonal means and interannual variability. Clim Dyn.

[4] Ruosteenoja K, Räisänen P (2013). Seasonal changes in solar radiation and relative humidity in Europe in response to global warming. J Clim.

[5] Bathiany S, Dakos V, Scheffer M, Lenton TM (2018). Climate models predict increasing temperature variability in poor countries. Sci Adv.

[6] Lobell DB, Schlenker W, Costa-Roberts J (2011). Climate trends and global crop production since 1980. Science.

[7] Thornton PK, van de Steeg J, Notenbaert A, Herrero M (2009). The impacts of climate change on livestock and livestock systems in developing countries: a review of what we know and what we need to know. Agric Syst.

[8] Chowdhury FR, Ibrahim QSU, Bari MS, Alam MMJ, Dunachie SJ, Rodriguez-Morales AJ, Patwary MI (2018). The association between temperature, rainfall and humidity with common climate-sensitive infectious diseases in Bangladesh. PLoS One.

[9] Sánchez-Molano E, Kapsona VV, Ilska JJ, Desire S, Conington J, Mucha S, Banos G (2019). Genetic analysis of novel phenotypes for farm animal resilience to weather variability. BMC Genet.

[10] Nguyen TTT, Bowman PJ, Haile-Mariam M, Pryce JE, Hayes BJ (2016). Genomic selection for tolerance to heat stress in Australian dairy cattle. J Dairy Sci.

[11] Carabaño MJ (2016). The challenge of genetic selection for heat tolerance: the dairy cattle example. Adv Anim Biosci.

[12] Rojas-Downing MM, Nejadhashemi AP, Harrigan T, Woznicki SA (2017). Climate change and livestock: impacts, adaptation, and mitigation. Clim Risk Manag.

[13] Martin JGA, Nussey DH, Wilson AJ, Réale D (2011). Measuring individual differences in reaction norms in field and experimental studies: a power analysis of random regression models. Methods Ecol Evol.

[14] West JW (2003). Effects of heat-stress on production in dairy cattle. J Dairy Sci.

[15] IPCC. Climate Change 2014: Synthesis Report. Summary for Policymakers. In: Pachauri RK, Meyer LA, editors. Climate Change 2014: Synthesis Report. Geneva: 2014. p. 2-26.

[16] Karakus F (2014). Weaning stress in lambs. J Int Sci Publ Agric Food.

[17] Kruuk LE, Clutton-Brock TH, Slate J, Pemberton JM, Brotherstone S, Guinness FE (2000). Heritability of fitness in a wild mammal population. Proc Natl Acad Sci U S A.

[18] Villanueva B, Kennedy BW (1990). Effect of selection on genetic parameters of correlated traits. Theor Appl Genet.

[19] McLaren A, Lambe NR, Brotherstone S, Conington J, Mrode R, Bünger L (2012). Investigation into the presence of genotype by environment interactions (G×E) in Scottish blackface lamb traits. Small Rumin Res.

[20] Misztal I, Tsuruta S, Strabel T, Auvray B, Druet T, Lee D (2002). BLUPF90 and related programs. Proceedings of 7th World Congress on Genetics Applied to Livestock Production.

[21] Lambe NR, Conington J, Bishop SC, Waterhouse A, Simm G (2001). A genetic analysis of maternal behaviour score in Scottish blackface sheep. Anim Sci.

[22] Gilmour AR, Gogel B, Cullis BR, Thompson R (2009). 2009 ASReml user guide release 3.0.

[23] Falconer DS, Mackay TFC (1996). Introduction to quantitative genetics.

[24] Sanchez-Molano E, Woolliams JA, Blott SC, Wiener P (2014). Assessing the impact of genomic selection against hip dysplasia in the Labrador retriever dog. J Anim Breed Genet.

[25] Raphaka K, Sánchez-Molano E, Tsairidou S, Anacleto O, Glass EJ, Woolliams JA, Doeschl-Wilson A, Banos G. Impact of Genetic Selection for Increased Cattle Resistance to Bovine Tuberculosis on Disease Transmission Dynamics. Front Vet Sci. 2018;5:237. 10.3389/fvets.2018.00237 PMC6174293 30327771

